# Draft Genome Sequences of Two *Streptomyces* Strains, MZ03-37^T^ and MZ03-48, Isolated from Lava Tube Speleothems

**DOI:** 10.1128/MRA.00576-20

**Published:** 2020-06-18

**Authors:** Jose L. Gonzalez-Pimentel, Valme Jurado, Bernardo Hermosin, Cesareo Saiz-Jimenez

**Affiliations:** a HERCULES Laboratory, Évora University, Évora, Portugal; b Instituto de Recursos Naturales y Agrobiología de Sevilla, Seville, Spain; Georgia Institute of Technology

## Abstract

Two *Streptomyces* strains were isolated from a lava tube in La Palma, Canary Islands. Genomic analyses suggest that the two strains could belong to the same species. Here, we report the draft genomes for these bacterial strains.

## ANNOUNCEMENT

Specific conditions in underground environments promote the development of new pathways for the survival of microorganisms (1). One of the most representative genera in these environments is *Streptomyces*. This genus is considered the main producer of bioactive compounds (2).

The microbiology of lava tubes in the Canary Islands began to be studied in recent years (3, 4). Two strains were isolated from two different samples, located in the same lava tube, using a sterilized scalpel and were placed in sterile tubes. MZ03-37^T^ was isolated from mucolite speleothems, whereas MZ03-48 was isolated from dark-brown biofilms. Samples were processed on the day of sampling, suspended in a saline solution, and inoculated on Petri plates with a nutrient agar medium with 0.2% glycerol. The study of taxonomic markers based on the 16S rRNA gene sequences and the five housekeeping genes described for multilocus sequence analysis (MLSA) (5) was carried out as described by Dominguez-Moñino et al. (6). The 16S rRNA analysis identified Streptomyces palmae CMU-AB204^T^ (98.70%), Streptomyces catenulae NRRL B-2342^T^ (98.28%), and Streptomyces ramulosus NRRL B-2714^T^ (98.28%) as the closest relatives for both strains. The MLSA revealed *S. catenulae* NRRL B-2342^T^ (*atpD*, *gyrB*, *recA*, and *trpB* genes) and *S. ramulosus* NRRL B-2714^T^ (*rpoB* gene) as the closest neighbors.

Isolation of genomic DNA from this bacterium was carried out using the Marmur method (7). Genomic DNA was sequenced using 250-bp paired-end reads on an Illumina HiSeq platform by means of a Nextera XT library preparation kit. Raw reads were adapter trimmed using Trimmomatic version 0.36 with a sliding window using a quality score cutoff of Q15 (8). The draft genome was assembled using SPAdes version 3.11.1 (9) with the flag “careful” to reduce the number of mismatches and short indels. Annotations were carried out using the NCBI Prokaryotic Genome Annotation Pipeline (PGAP) (10) and Sma3s (11) with the flag “uniprot” for the UniProt/Swiss-Prot database, whereas antiSMASH (12), with the detection strictness parameter in strict mode and all extra features on, was used for prediction of secondary metabolites. Sequencing and assembly statistics, as well as the NCBI PGAP results, are listed in Table 1.

**TABLE 1:** Statistics for genomes of strains MZ03-37^T^ and MZ03-48

Strain	No. of reads	Median insert size (bp)	Mean coverage (×)	No. of contigs	*N*_50_ (bp)	Size of largest contig (bp)	Genome size (bp)	G+C content (%)	Total no. of genes	No. of rRNAs	No. of tRNAs
MZ03-37^T^	4,091,770	277	191.3	814	16,301	160,358	6,995,846	72.2	6,313	15	65
MZ03-48	1,736,762	366	84.4	1,118	11,353	93,658	6,913,747	72.1	6,333	16	65

Relatedness between MZ03-37^T^, MZ03-48, and the closest species, as determined by 16S rRNA analysis and MLSA, was assessed by calculating the average nucleotide identity using BLAST (ANIb) and the average nucleotide identity using MUMmer (ANIm), by means of JSpeciesWS (13). The NCBI accession numbers for the closest relatives are SRID00000000 and JODY00000000 for *S. palmae* CMU-AB204^T^ and *S. catenulae* NRRL B-2342^T^, respectively, whereas the *S. ramulosus* NRRL B-2714^T^ genome was assembled for this study using the SRA data (accession number SRR7783857) (14), following the same methodology. Both strains, MZ03-37^T^ and MZ03-48, shared an identity of 99.98% for ANIb and ANIm. However, genome comparison analyses with the closest species showed scores below the recommended threshold (95%) for species delineation. These results suggest that strains MZ03-37^T^ and MZ03-48 belong to the same species, which differ from the closest type strains.

Sma3s predicted 83 and 82 genes probably involved in the synthesis of antibiotics in strains MZ03-37^T^ and MZ03-48, respectively. AntiSMASH predicted a total of 28 and 29 biosynthetic gene clusters in strains MZ03-37^T^ and MZ03-48, respectively. Promoting further studies of microbiology in subsurface environments could result in new biomolecules with biotechnological uses (15).

### Data availability.

The whole-genome shotgun projects for *Streptomyces* sp. strains MZ03-37^T^ and MZ03-48 have been deposited in DDBJ/ENA/GenBank under the accession numbers VKJP00000000 and VKLS00000000, respectively. The versions described in this paper are the first versions, VKJP01000000 and VKLS01000000, respectively. BioProject and raw data are available under the accession numbers PRJNA553665 and SRR10391217 for strain MZ03-37^T^ and PRJNA553134 and SRR10391142 for strain MZ03-48.
